# Canine angiostrongylosis in Sweden: a nationwide seroepidemiological survey by enzyme-linked immunosorbent assays and a summary of five-year diagnostic activity (2011–2015)

**DOI:** 10.1186/s13028-017-0351-7

**Published:** 2017-12-19

**Authors:** Giulio Grandi, Eva Osterman Lind, Roland Schaper, Erik Ågren, Manuela Schnyder

**Affiliations:** 1 Department of Microbiology, National Veterinary Institute, Ulls väg 2B, SE-756 51 Uppsala, Sweden; 2 Department of Biomedical Sciences and Veterinary Public Health (BVF), Swedish University of Agricultural Sciences, PO Box 7028, Ulls väg 26, SE-757 56 Uppsala, Sweden; 3 Bayer Animal Health GmbH, 51368 Leverkusen, Germany; 4 Department of Pathology and Wildlife Diseases, National Veterinary Institute, Ulls väg 2B, SE-756 51 Uppsala, Sweden; 5 Institute of Parasitology Vetsuisse Faculty, University of Zurich, Winterthurerstrasse 266a, 8057 Zurich, Switzerland

**Keywords:** *Angiostrongylus vasorum*, Antigen, Antibodies, Serology, Dog

## Abstract

**Background:**

For the first time in Sweden, *Angiostrongylus vasorum* was detected on the island of Sydkoster in foxes and dogs in 2003. After sporadic detection of the parasite in foxes in southern Sweden, the first positive canine faecal sample on the mainland was found in 2011. Since then a total of 2882 faecal samples have been analysed with the Baermann test at the National Veterinary Institute (SVA) during the years 2011–2015; 20 of them being positive. Contemporaneously, of over 525 fox necropsies, only three were found to be infected. To gather a more accurate knowledge of *A. vasorum* occurrence in Sweden, a large scale seroepidemiological survey was performed and totally 3885 serum samples from dogs were tested for both the presence of circulating antigens and of specific antibodies to *A. vasorum*.

**Results:**

In total, 0.10% (n = 4, 95% Confidence Intervals, CI 0.03–0.26%) of the dogs were positive for both antigen and antibodies, whereas 0.51% (n = 20, CI 0.31–0.79%) of the tested dogs were only antigen positive and 0.88% (n = 34, CI 0.61–1.22%) only positive for specific antibodies. Seropositive animals, as well as the majority of *A. vasorum*-positive faecal samples tested during the same period, were spread over central and southern Sweden. Annual prevalence of positive faecal dog samples and of necropsied *A. vasorum* positive foxes (coming from southern Sweden) varied from 0.3 to 0.9% (overall: 0.7%, CI 0.4–1.1%) and 0.0 to 1.4% (overall: 0.3%, CI 0.1–0.9%), respectively.

**Conclusions:**

The findings confirmed that *A. vasorum* has become established in various geographical areas of central and southern Sweden. Veterinarians and dog owners should be aware of the potential risks of infection in large areas of the country, since canine angiostrongylosis may be a fatal disease if left untreated.

## Background

*Angiostrongylus vasorum*, the French heartworm, is a nematode parasite of dogs. The worm resides in the right side of the heart and in the pulmonary arteries. The red fox (*Vulpes vulpes*) and other wild canids act as definitive hosts as do dogs, while intermediate hosts, terrestrial gastropod molluscs, are needed to complete the life cycle. Frogs and birds may be involved as paratenic hosts [1–4]. Although *A. vasorum* was reported from France in the 19th century [5], a more detailed characterization of the clinical features related to the infection as well as improvement of diagnostic methods mainly took place during the last 20 years.

Clinical signs may vary greatly but coagulopathy and lung lesions have been identified as the two main reasons for a potentially fatal outcome of the disease in dogs. Clinical manifestations of coagulopathy can range from subcutaneous haematomas, epistaxis to neurological signs depending on the site of bleeding. The lung damage is related to microinjuries and inflammatory processes taking place both when eggs of *A. vasorum* are caught in the lung capillaries and when the hatched larvae migrate to the air passages. Tissue injuries intensify and may lead to the classical textbook clinical presentation of the disease characterized by dyspnoea, exercise intolerance, coughing, followed by lethargy and weakness [6].

A more accurate description of the geographical distribution of *A. vasorum* can be obtained using detection methods characterized by high sensitivity and specificity; such a description can be useful to promote better prevention measures and to increase disease awareness in areas where the risk of infection is present [7]. Traditionally the diagnosis relies on the detection of first stage larvae (L1) from faeces, but the excretion of L1 only takes place when lung lesions have already developed and furthermore, the Baermann technique has limitations in sensitivity and specificity [7]. For these reasons, other techniques such as Polymerase Chain Reaction (PCR) and serology have been developed and tested on individuals and—in the case of serology—in large scale studies. *A. vasorum* PCR protocols using specific primers targeting the ITS-2 region are usually able to detect a single L1 of *A. vasorum* [8–10]. Enzyme-Linked Immunosorbent Assays (ELISAs) have also developed to detect circulating parasite antigens [11] or antibodies against the parasite in serum samples [12, 13]. The diagnostic approach usually follows or completes a clinical assessment of individual patients, while large scale surveys are conducted on large numbers of randomly collected samples from individuals, distinguishing between infected and non-infected animals and therefore giving information on the occurrence and prevalence of *A. vasorum* in a given population.

During the past decades, the endemic foci of infection have expanded and new ones have been reported. The relative importance of different factors related to this apparent spread is still unclear. Increased urbanization of the red fox acting as wildlife reservoir [14] and increased movement of domestic dogs both within and between countries [15] are certainly involved. Climatic changes influencing the distribution of intermediate hosts may also play a role [16]. Beside historically endemic areas, like southern France, part of the British Isles and Denmark, there is a list of countries in South, Central and East Europe where the presence of the parasite has consistently been recorded. Recently an increased prevalence of the parasite in foxes [17, 18] and dogs [19, 20] has been reported in e.g. the United Kingdom (UK) and in Italy. Moreover, new autochthonous cases have been described in other countries such as Belgium [21], Slovakia [22] and Serbia [23]. During the past years, it has been possible to obtain a more complete picture of parasite prevalence in dog populations through large scale serological studies carried out in some countries, for example in Germany and UK [24], Italy [25], Switzerland [26], Poland [27], Hungary [7], Slovakia [28], Bulgaria [29], Belgium [30] and Portugal [31].

In Scandinavia, *A. vasorum* was initially detected in 1983 in Denmark in a dog that had travelled several times to southern France [32]. In Danish foxes, *A. vasorum* was first reported in 1992 [33], and the prevalence in foxes in the same area (northern Zealand) rose from 48.7% in 1993 [34] to 90% in 2002 [35]. Despite a prevalence of 9.7% of *A. vasorum* infections was found in Danish dogs in 2004 [36], an overall prevalence of 2.2% was reported in hunting dogs in 2013 [37]. In Norway the parasite was identified for the first time in a red fox in March 2016 [38]. In Sweden, *A. vasorum* infection was first diagnosed in 2003, both in a dog and in a red fox (*Vulpes vulpes*) from the island of Sydkoster (Västra Götaland county) [39], and since then further individual cases of *A. vasorum* infections have been identified in dogs and red foxes on the mainland as well.

The aim of the present study was to determine the prevalence and geographical distribution of *A. vasorum* in Swedish dogs by using serological analyses and data from routine diagnostic activity performed at the National Veterinary Institute (SVA).

## Methods

### Prevalence study using ELISAs

Sera of 3885 dogs were collected from the Clinical Chemistry Laboratory (CCL) of the University Animal Hospital (UDS-SLU, Uppsala) and from the SVA. Sera from CCL (n = 1212) were collected from February to April 2014, either from dogs attending the University Animal Hospital (n = 495) or were sent to CCL from other veterinary clinics of the country (n = 717). Sera collected from the SVA (n = 2674) were submitted from veterinary clinics during 2013 for the analysis of tick-borne bacterial pathogens such as *Anaplasma phagocytophilum* and *Borrelia* spp. For each sample the postal code of the residence of the dog owner was recorded. No other data could be retained due to confidentiality issues.

An aliquot of the samples was sent to the Institute of Parasitology, Vetsuisse Faculty, University of Zurich, Switzerland, for analysis for the presence of circulating *A. vasorum* antigens using monoclonal (mAb Av 56/1/2) and polyclonal antibodies in a sandwich ELISA with a sensitivity of 95.7% and a specificity of 94.0%, as previously described [11]. In brief, tests were performed in 96-well microtiter plates (Maxisorp, Nunc Roskilde, Denmark) coated overnight at 4 °C with 2.5 µg/mL of mAb Av 56/1/2 that had been obtained by the fusion of spleen cells of mice immunised against adult *A. vasorum* excretory/secretory (E/S) antigen with myeloma cells, based on the protocol of de StGroth and Scheidegger [40]. Plates were then washed and saturated for 30 min at 37 °C with PBS-Tween-20 (PBS-T). Serum dilutions (100 µL/well, 1:2 in PBS–T) were incubated for 1 h at 37 °C. After further washing steps, hyperimmune polyclonal rabbit serum (100 µL/well) from rabbits also immunised with adult *A. vasorum* E/S antigen was added in a concentration of 1:500, incubated for 1 h at 37 °C and plates again washed before adding 100 µL/well goat anti-rabbit IgG conjugated to alkaline phosphatase and diluted in PBS–T (1:2500). After the repetition of the incubation and washing steps, 100 µL/well of substrate (*p*-nitrophenyl phosphate) were added. Absorbance values were read at 405 nm (OD405) with a Multiscan RC ELISA reader (Thermo Labsystems, Helsinki, Finland). The ELISA has been shown to detect circulating antigens starting at 5 weeks post infection (wpi) and at 10 wpi virtually all dogs are persistently positive. After anthelmintic treatment, antigens can be detectable until 9 weeks post treatment [11]. Additionally, a sandwich ELISA (sensitivity 81.0%, specificity 98.8%) using *A. vasorum* adult somatic antigen purified by monoclonal antibodies (mAb Av 5/5) was used for specific antibody detection [14]. Briefly, plates were coated overnight at 4 °C with *A. vasorum* adult somatic antigen (5 µg/mL), washed and saturated. Sera were used in a standard dilution of 1:200 in PBS-T with 10% foetal calf serum; 100 µL diluted sera/well were incubated at 37 °C in a humid chamber for 1 h. After washing steps, specific affinity-purified, alkaline phosphatase conjugated goat anti-dog IgG (γ) antibodies were used at a dilution of 1:250 in PBS-T (100 µL/well). Substrate was added after the washing steps and absorbance values obtained as described above. The ELISA has been shown to detect antibodies from 3 to 6 wpi and to remain positive as long as the animal is infected. After anthelmintic treatment, antibodies may remain detectable until 9 weeks post treatment [13].

All test runs included a background control, a conjugate control, three positive control sera from three experimentally infected dogs and two negative control sera from uninfected dogs, as previously described [11, 13]. Test thresholds were regionally determined with 300 randomly selected samples based on the mean value of optical density (A_405_ nm) plus 3 standard deviations.

The collected data were embedded into a geographical information system (GIS) using the program RegioGraph 10 (GfK GeoMarketing, Bruchsal, Germany) to visualize the regional distribution of *A. vasorum* antigen- and/or antibody-positive samples. The locations of positive samples were displayed on maps with administrative and postcode boundaries based on the three-digit postcodes of Sweden as points of reference.

Excel 2007 for Windows (Microsoft Corporation, Redmond, USA) was used to calculate the prevalence values and the 95% confidence interval (CI) of prevalence values.

### Data from routine diagnostic activity, 2011–2015

#### Dogs

Faecal samples (Table 2) submitted to the SVA by veterinary clinics or private dog owners were tested with a modified larval recovery method [41]. The SVA requires at least three samples from three different droppings collected over 3 days from each dog in order to perform the analysis. All samples originated from dogs showing clinical signs, either respiratory or other clinical signs compatible with *A. vasorum* infection, and therefore cannot be considered representative of the general canine population.

#### Red foxes

Red foxes (Table 2) received at the SVA for diagnostic necropsies or submitted by hunters for pathogen screening were examined specifically for *A. vasorum* and lungworms by visual inspection of the cardiovascular and respiratory systems. During 2011, 3000 hunted red foxes were submitted for the national *Echinococcus multilocularis* surveillance program. Over 500 heart and lung sets were examined for parasites that year. A joint database was created in order to produce Table 2.

## Results

**Fig. 1:**
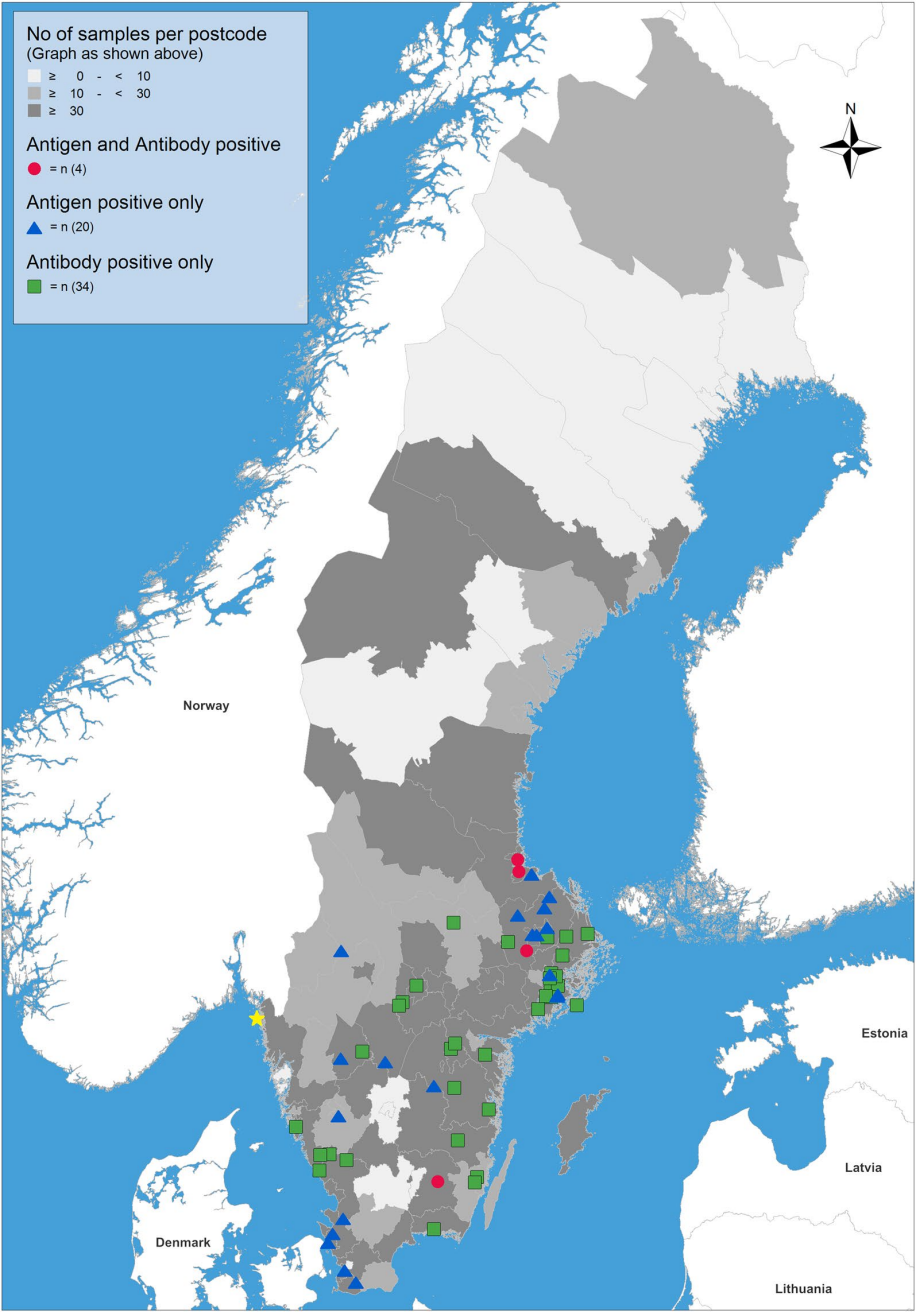
Detection of *Angiostrongylus vasorum* circulating antigens and specific antibodies in serum samples from 3885 dogs from Sweden. Dark grey areas represent the origin of the tested dog sera. The first place of finding of *A. vasorum* in Sweden (island of Sydkoster) is marked with a yellow star

A total of 0.10% (n = 4, 95%, CI 0.03–0.26%) of the dogs were positive in both ELISAs, while 0.51% (n = 20, CI 0.31–0.79%) were antigen positive only and 0.88% (n = 34, CI 0. 1–1.22%) were positive for specific antibodies only (Table 1). The geographical distribution of positive samples is shown in Fig. 1. Samples positive in both tests were found only in the counties of Gävleborg, Uppsala and Kronoberg (Fig. 1).

**Table 1:** Serological results of 3885 dog serum samples from Sweden tested for the presence of circulating antigens of *Angiostrongylus vasorum* and of specific antibodies to *A. vasorum*

	Positive samples (n)	%	95% confidence intervals
Antibody-positive	38	0.98	0.69–1.34
Antibody-positive only	34	0.88	0.61–1.22
Antigen positive	24	0.62	0.40–0.92
Antigen positive only	20	0.51	0.31–0.79
Antibody and antigen positive	4	0.10	0.03–0.26

Endemic canine cases of *A. vasorum* were diagnosed once in 2011 and 2012, respectively. Subsequently, the parasite was sporadically found (4–9 times per year). A progressively increasing number of faecal samples was submitted to the SVA throughout this period.

Detailed figures on the number of examined faecal samples as well as the number of foxes necropsied at the SVA between 2011 and 2015 are provided in Table 2.

**Table 2:** Results from the diagnostic activity concerning *A. vasorum* related methodologies carried out at the SVA between 2011 and 2015

	Year					Total
	2011	2012	2013	2014	2015	
Groups	Numbers					
Dogs, Baermann tested samples	298	355	504	693	1032	2882
*A. vasorum* positive canine faecal samples (%)	1 (0.3; 0.0–1.9)	1 (0.3; 0.0–1.6)	4 (0.8; 0.2–2)	5 (0.7; 0.2–1.7)	9 (0.9; 0.4–106)	20 (0.7; 0.4–1.1)
County of origin*	M	O	M, S, K, Y	AB, C, D, H, M	AB, BD, E, O, M, Y, Z	–
Red foxes necropsied	525	88	164	70	100	947
*A. vasorum* positive foxes (%)	1 (0.2; 0–1.1)	0 (0.0; 0.3.3)	1 (0.6; 0.0–3.4)	1 (1.4; 0.0–7.7)	0 (0; 0.0–3.0)	3 (0.3; 0.1–0.9)
County of origin*	G	–	O	O	–	

Codes for Swedish counties: AB: Stockholm, BD: Norrbotten; C: Uppsala, D: Södermanland, E: Östergötland, G: Kronoberg; H: Kalmar, K: Blekinge, M: Skåne; O: Västra Götaland; S: Värmland; Y: Västernorrland; Z: Jämtland

## Discussion

The first Swedish fox being positive for *A. vasorum* was identified in 2003 on the island of Sydkoster, in the county of Västra Götaland (see Fig. 1). Six years later, in the beginning of 2009, the parasite was found in the lungs of four out of 269 foxes examined within an *E. multilocularis* surveillance program; again, the positive animals originated from Sydkoster. After this finding, a targeted survey aimed at finding other foci of *A. vasorum* was performed in 2009 on 137 foxes from south-western Sweden. Eight foxes were found to be infected, of which seven were from Sydkoster (64% of the foxes collected on this island within the survey) and one from the municipality of Osby (county of Skåne), which was the first *A. vasorum* case described on mainland Sweden [42]. Even though many red foxes have been necropsied thereafter, the parasite has been identified only three additional times, in 2011, 2013 and 2014 (Table 2). The fox that tested positive in 2011 is particularly interesting because it originated from the municipality of Kalvsvik (county of Kronoberg), in the same area where one of the four dogs being both positive for antibodies and antigen was found. No double positive samples were identified in the county where the majority of positive foxes were previously found (Västra Götaland).

The implications of positive results and the performance of the two assays used in the present study have been evaluated independently by determining sensitivities and specificities [11, 13]. In the present study, the number of sera positive for antibody or antigen detection only could indicate an apparently low correlation between the two ELISAs used. However, when combined, these test parameters change: using the OR rule (the combined results are considered positive if either individual test is positive), sensitivity increases by loss of specificity, while using the AND rule (the combined results are considered positive only if both individual tests are positive), specificity reaches 99.9%, while sensitivity decreases to 82.0% [24]. Importantly, these values are influenced by the prevalence of *A. vasorum* within a determined population: the positive predictive values of the serological tests, represented by the proportion of positive test results that indicate a truly positive sample, is highest with the AND rule and this may of particular value in study areas where little is known on the occurrence of the parasite: reliability of a positive sample is high. However, at low prevalence, such high positive predictive values are reached at the expense of low sensitivity [24].

Therefore, a combined positive result indicate a dog that harbours an active *A. vasorum* infection inducing the production of antibodies; it has higher specificity than that of a single positive test (for antigen or antibody detection), although sensitivity is lower. However, as such a test combination is reflected by high positive predictive values, its use is of particular interest in areas of expected low endemicity, as it may occur in countries with relatively new introduction of the parasite like in Sweden.

This is supported by large scale studies from Germany [24] and Poland [27]: applying similar methodologies, seroprevalences of *A. vasorum* were higher than the ones detected in Swedish dogs. Intermediate prevalence figures have been recorded in Italy [25] and in the UK [24], whereas the highest prevalence so far was recorded in Hungary [7]. The seroprevalence of antibody-positive dogs in Sweden was the lowest recorded so far. Dogs positive for antibodies only may have acquired the infection recently, i.e. 3 weeks before (while antigen is detected earliest 5 wpi), but they also could be parasite-free, i.e. after anthelmintic treatment or spontaneous clearance of infection. The data confirmed that the level of exposure to the parasite in Sweden is still quite low. This is in accordance with the extremely low number of infected foxes detected so far, which are considered as a reservoir contributing to the establishment of the parasite [26, 33], as supported by constantly higher prevalences in foxes is than in dogs in corresponding areas [43]. In this context it needs to be added that the difficulty to identify positive foxes with low worm burdens combined with low disease awareness in an early phase of investigations may have contributed missing positive foxes, and that dissection may not be considered as a gold standard [44].

Another indirect demonstration of the low endemic presence of the parasite in Sweden is given by the results of faecal examinations performed at the SVA, presupposing that samples obtained from clinically ill dogs do not represent the general canine population. After the first description of an autochthonous case in a dog in 2003, the first dog was not diagnosed until 2011 by a Baermann analysis. Because of the relatively common (8–9%) occurrence of *Crenosoma vulpis,* the fox lungworm, Baermann examinations are being regularly performed at the SVA. After the first report of *A. vasorum* in Sweden the number of examinations have steadily increased, however, despite this increase, the number of *A. vasorum* positive findings has remained low (Table 2).

Detection of the parasite both in foxes and dogs in Denmark and Sweden, but only one recent finding in a red fox in Norway and still no occurrence of the parasite in Finland suggest that *A. vasorum* may have been introduced from Denmark and/or Germany. Southern Sweden is often visited by tourists from these countries, especially during the summer, and they are often accompanied by their dogs. Another interesting aspect is the partial overlapping of geographical distribution of samples positive by serology with samples positive at coprological examination (data not shown). In fact, all the positive samples (faecal or serum samples) originated from geographic areas that in a prediction model have been shown to be suitable for the establishment of the parasite [16], although it cannot be excluded that some of the positive dog cases found in the present study are not endemic but may have been imported from other endemic foci instead. The presence of an abundant fox population especially in agricultural regions, coinciding with the well-established circulation of lungworms (*C. vulpis*) that use molluscan intermediate like *A. vasorum*, is a situation with high risk for spreading of the parasite in Scandinavia. This is for instance supported by data from neighbouring Denmark, where the occurrence of the parasite in foxes has increased rapidly during the last 20 years.

## Conclusions

The results confirmed that *A. vasorum* is endemically present in Sweden: not limited to restricted areas such as the Island of Sydkoster and the municipality of Osby in Scania, but also in foci in widespread areas of the country. Since suitable conditions for the establishment of *A. vasorum* apparently exist in southern Sweden and potentially also in other areas of Sweden, disease awareness among practitioners, dog owners and hunters, and an according strategical deworming management is recommended, despite the currently low prevalence. This may be particularly important in the case of dogs living or visiting highly endemic areas, in order to avoid further introduction of the parasite into new areas (with the potential to create new foci), and to prevent clinical disease due to angiostrongylosis. Regular deworming should be considered in dogs which belong to “high risk categories”, such as young dogs and/or dogs having free roaming activity that show risk behaviour like indiscriminate ingestion of items from their environment.
